# A study of self-precaution against the background of the COVID-19 pandemic from the perspective of risk perception attitude theory and social support

**DOI:** 10.1186/s12889-021-11597-9

**Published:** 2021-08-10

**Authors:** Ruo-Nan Wang, Yue-Chi Zhang, Rang-Ke Wu, Bei Li, Chuang-Wei Li, Bo-Tao Yu, Yi-Li Zhang

**Affiliations:** 1 School of Health Management, Southern Medical University, Guangzhou, 510515 China; 2 Bussiness school, University of Aberdeen, Aberdeen, UK; 3 School of Foreign Studies, Southern Medical University, Guangzhou, 510515 China

**Keywords:** Risk perception attitude theory, Social support, Self-precaution, COVID-19

## Abstract

**Background:**

In this research, the factors that influence the self-precautionary behavior during the pandemic are explored with the combination of social support and a risk perception attitude framework.

**Methods:**

An online survey was conducted among 429 members to collect information on demographic data, social support, perceptions of outbreak risk, health self-efficacy, and self-precautionary behaviors with the guide of the Social Support Scale, the COVID-19 Risk Perception Scale, the Health Self-Efficacy Scale and the Self-precautionary Behavior Scale.

**Results:**

The research shows that among the three dimensions of social support, both objective support and support utilization negatively predict risk perception, while subjective support positively predicts health self-efficacy; health self-efficacy and risk perception significantly predict self-precautionary behavior; the relationship between risk perception and self-precautionary behavior is significantly moderated by health self-efficacy.

**Conclusions:**

The combined influence of social capital and risk perception attitudinal frameworks on self-precautionary behavior is highlighted in this study, with the relationship between the public’s risk perception, health self-efficacy, and self-precautionary behavior intentions examined against the background of coronavirus disease 2019 (COVID-19). These findings contribute to understanding the impact of social capital factors on risk perception and health self-efficacy, which provides insight into the current status and influencing factors of the public’s precautionary behavior and facilitates early intervention during a pandemic.

## Background

The outbreak of coronavirus disease 2019 (COVID-19) has posed a huge challenge to the production, livelihood and health security of people around the world. Due to its severity, the World Health Organization (WHO) identified the COVID-19 outbreak as a Public Health Emergency of International Concern on January 30, 2020, and declared it as a pandemic on March 11, 2020 [1]. By April 13, 2021, a total of 90,447 cases in China have been confirmed, including 85,513 cured and discharged, and 4636 dead [2]. By 5 o ‘clock on April 13, 2021, a total of 136,291,755 cases and 2,941,128 deaths have been confirmed in the whole world [3]. The highly infectious nature of COVID-19 makes the disease quite alarming in terms of the total number of deaths, in spite of its low death rate (compared with SARS and MERS epidemics) [4]. Epidemic prevention and control require national emergency response and effective measures, effectual response by public health resources, full cooperation, active protection, and conscious prevention and control by individuals in society. Most importantly, the implementation of non-medical measures for self- precaution (such as washing hands regularly, wearing masks, and keeping interpersonal distance) is directly related to the overall situation of epidemic prevention and control.

The role played by self-precaution in the prevention and control of major public health emergencies has been extensively studied. The findings of Wise et al. suggest that personal perceived risk is correlated with self-precautionary behaviors during a pandemic, and understanding the mechanisms underlying the correlation between the two can be conducive to its early intervention [5]. Loeb and Delaney et al. in MBIDs (mosquito-borne infectious disease) disease study conclude that the adoption of self-precautionary behaviors (SPBs) is the most effective prevention mechanism for reducing the risk of MBID [6, 7]. Omodior et al. explored the role of perceived risk for mosquito-borne infectious disease in international travelers’ self-precaution in the United States. They conclude that the degree of perception of Zika disease among international travelers in the United States is positively associated with adopting three self-precautionary behaviors [8]. Other researchers have further examined issues related to self-precaution [9–11].

The risk perception attitude (RPA) framework has been widely adopted to predict the public’s motivation for self-precaution [12]. Individuals take or intend to take precautionary measures to protect themselves against certain diseases. Studies have been conducted to validate the predictability of the RPA framework for individual precautionary behaviors [12–14]. An important motivation of this research is to further expand the scope of RPA framework by studying whether individual willingness for self-precaution is really effective during the pandemic.

On the one hand, pandemic outbreaks are a challenge for the government as well as the public. During the occurrence of a disease, the government will adopt various prevention and control strategies, such as city closure, home quarantine, and shutdown of work and production, in order to control the harm caused by the disease. The implementation of these measures will undoubtedly bring great stress and anxiety to the public [15]. Even though they perceive the severity and susceptibility of the disease, the public may resist taking self-precautionary measures based on their anxiety. Therefore, individual risk perception and precaution may be negatively correlated as a result.

On the other hand, members of the public who have the ability and confidence to deal with their own health problems may be more inclined to believe that self-precautionary behavior can help improve their health status and reduce the rate of disease infection, thus reinforcing their willingness to take self-precautionary behaviors. Thus, individual perceptions of disease risk and health self-efficacy during the course of a pandemic may also have an impact on self-precaution.

To explain self-precaution, other theories have been used in previous studies, like the Health Belief Model (HBM) [16]. The main distinction between RPA and HBM is that RPA studies the impacts of risk perception and health self-efficacy on individuals’ intentions to engage in precautionary behavior, whereas HBM focuses on the impacts of attitudes and subjective norms on individual behaviors, more specifically focusing on the relationship between health behavior and health service utilization [17]. Since attitude has been found to have a significant effect on behavior in the existing research literature [18, 19] and numerous empirical tests of this theoretical model have been conducted in the existing research literature [20–23], it may be fruitless to further explore this research from the perspective of HBM theory. Besides, more advanced information technology may be employed to intervene into self-precautionary behaviors during the course of the pandemic. However, due to the limited knowledge about the disease itself as well as its preventive measures, the information received by the public through various media may lead to irreversible consequences if not correctly understood. Thus, perceived disease risks can influence the public’s precautionary behavior. Therefore, we have adopted the RPA theoretical perspective in the current study rather than other theories like HBM.

Individual personality traits, such as self-efficacy associated with previous experiences, may influence individuals’ risk perception [24]. Social support, a core concept of health and well-being, has been proposed and shown to have a positive impact on health outcomes. Paykani et al. revealed that social support provided to the public by family, media, and policymakers during the course of a pandemic would help individuals comply with social distancing orders and thus reduce disease infection rate [11]. Social support has been proved to be an influential factor in risk perception, with a negative correlation between them [25, 26]. For example, a study of pregnant women in late pregnancy during the COVID-19 conducted by Chongyu Yue et al. shows that the social support received by pregnant women negatively influences perceived risk, which mediates the relationship between social support and anxiety [27]. Besides, the relationship between the social support received by individuals and health self-efficacy has been extensively studied [28]. For example, Rashid AA et al. proves a significant correlation between social support and patients’ self-efficacy in handling medications in a study of factors influencing health behaviors in patients with type 2 diabetes [29]. Thus, social support can be regarded as a predictor of risk perception and health self-efficacy.

There are several other control variables such as age, gender, education, and marriage that influence the public’s self- precautionary behavior during a pandemic [10, 30]. For instance, people’s ability to process the large amount of information released by the media about COVID-19 varies depending on the individual’s education degree. For most self-precautionary behaviors (e.g., avoiding parties, wearing masks, and increasing hand hygiene), studies have shown that people with low education levels are less likely to take these measures, and they are more likely to undertake entirely unprotective behaviors [10]. Therefore, in the present study, we have also taken the effects of control variables into consideration.

## Theoretical background and hypothesis development

### The RPA framework

The RPA framework has been widely used to predict the public’s motivation for self-precaution, showing the impacts of risk perception and self-efficacy on individual health behaviors [12]. Risk perception refers to the psychological state that individuals exhibit as a result of perceiving external risks, reflecting their responses to the external environment beyond their intuitive judgments and subjective feelings [31]. Perceived self-efficacy, on the other hand, refers to “people’s belief in their ability to generate a specified level of performance that affects the events that affect their lives” [32]. This study is designed to investigate the self-precautionary behavior of the public during a pandemic. Therefore, the construct “health self-efficacy” is used in the study, and it means “an individual’s belief that they are confident in managing their own health.” [33].

The public must be confident that they can overcome the negative health outcomes that may result from the anticipated risks. It has been established that the belief that individuals can successfully engage in desired health behaviors significantly influences individual precautionary behaviors [28, 29]. As health self-efficacy increases, the propensity to engage in self-precautionary behaviors will increase.

Risk perception is considered as a reliable predictor of individuals’ adoption of precautionary behaviors, which is also specified in various health behavior theories, such as the health belief model [33] and the protective motivation theory [34]. However, the causal relationship between risk perceptions and self-precautionary behavior is demonstrated to be relatively weak. Some researches [35–37] present a positive correlation between perceived risk and precautionary behavior. Yet others [38, 39] show a negative correlation. Chaffee and Roser [40] proved that a high level of risk perception would cause excessive fear and anxiety and, therefore, inhibits action, which has also been confirmed by empirical studies [41]. COVID-19, which has been ongoing for a long time, increases anxiety levels when the public perceive a particular risk of disease [15, 42–44], which in turn leads to a weakening of personal intentions to act precautionarily. Consequently, the following hypotheses are formulated in this study.

- Hypothesis 1 (H1). *Risk perception is negatively associated with self-precautionary behavior.*
- Hypothesis 2 (H2). *Health self-efficacy is positively associated with self-precautionary behavior.*
- Hypothesis 3 (H3). *Health self-efficacy plays a moderating role in the effect of risk perception on self-precautionary behavior.*

### Social support

Social support refers to the mutual material and moral assistance of individuals, as well as the exchange of material and moral resources [45]. Numerous empirical studies have been carried out to show that social support is an essential predictor of individual precautionary behavior [11, 46]. Shuiyuan Xiao et al. [47] classifies social support in three dimensions: subjective support, objective support, and support utilization, which have been validated in relevant empirical studies about Chinese population [27, 48, 49]. The present study complies with the findings of Shuiyuan Xiao et al. and tests these dimensions because they are appropriate and representative for Chinese population as the subject of the survey.

The associations between social support, health self-efficacy, and risk perception have been extensively studied by scholars. Social support substantially affects risk perception, and they are negatively correlated with each other [25–27]. Social support can positively influence health self-efficacy [28, 46]. During the course of a pandemic, people receive support from various sources, including some of the government’s preparedness measures, such as the provision of anti-pandemic supplies and timely information about the outbreak through official social media sites. When the public perceives social support through various channels, their pressures and uncertainties will be reduced, and thus perceived risks will be lowered as they receive real help and feel the love and trust from others. In addition, various types of social support achieved through official channels can help to send more information about the disease to the public, who will be more confident in coping with the pandemic, and therefore health self-efficacy will increase. Some studies have proved it [50, 51]. On the basis of these findings, we hypothesize that all three dimensions of social support have a positive effect on health self-efficacy and negative effect on risk perception, i.e.

- Hypothesis 4a (H4a). *Objective support is negatively associated with risk perception.*
- Hypothesis 4b (H4b). *Subjective support is negatively associated with perception.*
- Hypothesis 4c (H4c). *Support utilization is negatively associated with risk perception.*
- Hypothesis 5a (H5a). *Objective support is positively associated with health self-efficacy.*
- Hypothesis 5b (H5b). *Subjective support is positively associated with health self-efficacy.*
- Hypothesis 5 c(H5c). *Support utilization is positively associated with health self-efficacy.*

**Fig. 1:**
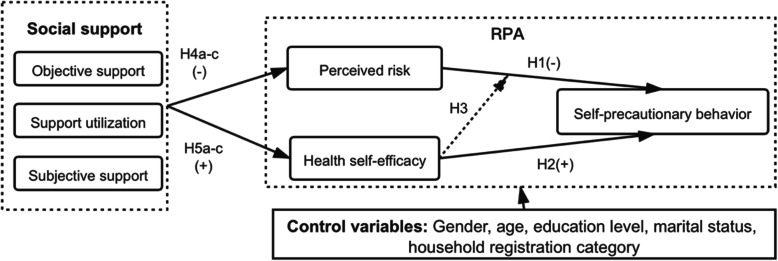
Research model

Figure 1. shows the research model.

## Methods

### Measurement

The final questionnaire used in our study contains two parts. The first part is a survey of demographic information, consisting of gender, age, education level, marital status, and household registration category. The second part consists of four subscales: social support, epidemic risk perception, health self-efficacy, and self-precautionary behavior. More information of these constructs is presented in Table 1.

**Table 1:** Research structures

Structure	Definition	Number of items measured	Source
Risk perception (PR)	The psychological state that an individual exhibits results from perceiving external risks, reflecting the individual’s response to the external environment beyond intuitive judgments and subjective feelings.	9	[31]
Health self-efficacy (HSE)	An individual’s confidence in managing his or her own health, reflecting the individual’s beliefs.	4	[32]
Objective support (OS)	Material direct assistance, and the presence and participation of social networks, group relations.	3	[47]
Subjective support (SS)	The individual’s emotional experience and satisfaction of being respected, supported and understood in society, which is closely related to the individual’s subjective feelings.	4	[47]
Support Utilization (SU)	The extent to which the individual utilizes the support received.	3	[47]
Self-precautionary Behavior (SPB)	The self-protective actions individuals take in response to perceived risks.	10	[56]

### Social support subscale

The Social Support Rating Scale [47] developed by Shuiyuan Xiao is used in this study to measure people’s social support during the pandemic. The scale consists of ten entries and three dimensions of objective support (sum of ratings for entries 2, 6, and 7), subjective support (sum of ratings for entries 1, 3, 4, and 5), and support utilization (sum of ratings for entries 8, 9, and 10). The 4-point Likert-type scoring method is used for all questions except questions 6 and 7, which are scored based on the number of sources of social support. The scale is totally scored between 12 to 66, and its three dimensions, eg., objective support, subjective support, and support utilization between 1 ~ 22, 8 ~ 32 and 3 ~ 12, respectively [52]. Good reliability and validity have made the scale extensively used [53]. The overall structural fit of the scale in this study is effective: χ^2^/df = 1.753, NFI = 0.974, IFI = 0.989, CFI = 0.989, RFI = 0.952, RMSEA = 0.042. In this study, the *Cronbach’s α* coefficients of the three dimensions of the scale are 0.81, 0.84 and 0.87 respectively. The coefficient of *Cronbach’s α* total scale is 0.80.

### Epidemic risk perception subscale

The COVID-19 Risk Perception Scale [54] developed by Juzhe Xi et al. is used in the current study. The scale consists of nine questions scored by Likert 4, 5 and 6 points scale with the total scale score range of 9 ~ 47 and higher scores indicating higher risk perception. It has good reliability with a KMO value of 0.94 and a satisfactory structural fit: χ^2^/df = 2.200, GFI = 0.970, AGFI = 0.950, NNFI = 0.970, CFI = 0.980, RMR = 0.030, RMSEA = 0.060. In this study, the scale structure fit well: χ^2^/df = 3.312, NFI = 0.971, IFI = 0.980, CFI = 0.980, RFI = 0.945, and RMSEA = 0.073, with the *Cronbach’s α* coefficient of the scale being 0.85.

### Health self-efficacy subscale

The five-item scale [55] developed by Lee et al. is adapted and then employed to assess health self-efficacy in the current study after excluding the item of “I can keep myself healthy and pay close attention to my health condition”. The remaining four items show acceptable construct validity and high internal consistency: χ^2^/df = 1.380, RMSEA = 0.030, CFI = 0.998, IFI = 0.998, RFI = 0.982, NFI = 0.994. The *Cronbach’s α* coefficient for this scale in this study is 0.71. The total score of the adapted scale was 4 ~ 20 by a 5-point Likert scale.

### Self-precautionary behavior subscale

The entries in this section of the Self-precautionary Behavior Strengths and Weaknesses Rating of the Public Knowledge and Behavior Questionnaire [50] related to the COVID-19, developed by Chichen Zhang et al., are used in the study, containing a total of 10 behavioral measures. The behaviors are divided into five stages: unconscious stage, behavioral interruption, conscious stage, planning stage, and behavioral stage, with the scores ranging from 0 to 4. The total score of self-precautionary behavior, calculated by summing each entry score, ranges from 0 ~ 40, with higher scores indicating better self-precautionary behavior. The structure of the scale in this study is well fitted: χ^2^/df = 2.657, NFI = 0.969, IFI = 0.981, CFI = 0.980, RFI = 0.947, and RMSEA = 0.062. The *Cronbach’s α* coefficient for this scale in this study is 0.85.

### Data collection

All the scales including the demographic information table were used for the survey online by uploading them onto the Questionnaire Star, a Chinese online survey platform, (https://www.wjx.cn), on which a link to the scales was generated for data collection via WeChat and Weibo, the most widely used social media platforms in China. With the use of snowball sampling, 458 respondents completed the questionnaire online within a week between 12 and 18 April, 2021. The final valid responses to the survey are 429, with the valid response rate of 93.67%. Among the interviewees, 69% are female and approximately 79.7% are under the age of 30, and 76.7% have bachelor’s degree, as is shown in Table 2.

**Table 2:** Sample characteristics (*n* = 429)

Variables		Frequency	Percentage (%)
Gender	Male	133	31.0
	Female	296	69.0
Age	18 ~ 30	342	79.7
	31 ~ 45	62	14.5
	46 ~ 60	22	5.1
	61 years old and above	3	0.7
Education level	Primary school	6	1.4
	Junior high school	36	8.4
	High school	29	6.8
	Specialized	29	6.8
	Bachelor’s degree and above	329	76.7
Marital status	Married	99	23.1
	Unmarried	330	76.9
Household registration category	Agricultural household registration	247	57.6
	Urban household registration	182	42.4

### Statistical analysis

SPSS 26.0 and the SPSS macro program process plug-in prepared by Hayes are used to process the data for statistical analysis. For this research, the *P*-value under 0.05 is considered statistically significant.

## Results

### Common method Bias test

This research conducts data collection with the self-report method, which may be subject to the problem of common method bias. On the one hand, according to the suggestion of Hao Zhou and Lirong Long [57], the procedure is controlled by means of responding anonymously, the tester explaining the sentences that are likely to cause confusion, and explaining to the study participants that the data obtained are for scientific research only. On the other hand, Harman’s one-way test is used for statistical control before the analysis of data, which means that the items of all variables are subjected to unrotated principal component factor analysis. The results show that six factors with characteristic roots greater than one are obtained after factor analysis, and the variance explained by the first factor is 23.13%, which is far less than the critical value of 40%. Therefore, the effect of common method bias on the results of this study can be ruled out.

### Correlation analysis

The correlations of the study variables are analyzed by Pearson correlation, and the results are shown in Table 3. OS, SS, SU, PR, HSE, and SPB are in pairwise correlation with each other, which provides the basis for further testing the research hypotheses.

**Table 3:** Descriptive statistics and correlation analysis (*n* = 429)

Construct	M	SD	1	2	3	4	5	6
0S	2.750	0.850	1					
SS	4.041	0.870	0.561***	1				
SU	2.696	0.576	0.852***	0.591***	1			
SPB	3.028	0.570	−0.188***	0.121*	−0.191***	1		
HSE	3.890	0.652	0.383***	0.730***	0.438***	0.242***	1	
PR	2.588	0.772	−0.431***	−0.294***	− 0.426***	− 0.332***	−0.231***	1

*p<0.05, **p<0.01, ***p<0.001

### Verification results of structural equation model

The structural equation model is tested by means of regression. The model contains six potential constructs, namely, OS, SS, SU, PR, HSE, and SPB. The effects of the main factors are examined first (Model 1). Subsequently, control variables such as gender, age, education, marital status, household registration category and the interaction of HSE and PR (Model 2) are added to examine control and moderating effects. The multiple regression results of PR, HSE and SPB are shown in Table 4 and the results of the hypothesis testing are shown in Table 5. The findings indicate that a significant correlation exists between the factors of HSE and PR and SPB. To be specific, SS positively affects HSE while OS and SU negatively affect PR. The significance of the path coefficients of the control variables indicates that gender can influence SPB; people with different educational backgrounds have different levels of HSE; furthermore, the relationship between PR and SPB can be adjusted significantly by HSE.

**Table 4:** Results of multiple regression analysis

Independent variables	Perceived risk		Health self-efficacy		Self-precautionary Behavior	
	Model 1	Model2	Model 1	Model 2	Model 1	Model 2
Independent variables						
HSE					0.187^***^	0.209^***^
PR					−0.284^***^	− 0.210^***^
HSE × PR						−0.332^***^
Social support						
OS	−0.240^**^	− 0.240^**^	− 0.115	−0.117		
SS	−0.044	−0.041	0.735^***^	0.725^***^		
SU	−0.195^*^	−0.198^*^	0.101	0.118		
Gender		−0.080		−0.013	0.093^*^	0.077
Age		0.078		−0.018	0.076	0.079
Education level		0.081		−0.096^*^	0.104	0.062
Marital status		0.038		0.046	0.021	0.064
Household registration category		0.028		−0.036	−0.057	− 0.053
R^2^	0.199	0.213	0.537	0.545	0.156	0.259
ΔR^2^		0.014		0.007		0.103
ΔF		1.464		1.372		58.356^***^

Variables in the model are standardized; *p < 0.05, **p < 0.01, ***p < 0.001.

**Table 5:** Hypothesis testing results

Hypothesis	Pathway	Coefficient^**a**^	Result^**b**^
H1	PR → SPB	−0.284^***^	Y
H2	HSE → SPB	0.187^***^	Y
H3	HSE × PR → SPB	−0.332^***^	Y
H4a	OS → PR	−0.240^**^	Y
H4b	SS → PR	−0.044	N
H4c	SU → PR	−0.195^*^	Y
H5a	OS → HSE	−0.115	N
H5b	SS → HSE	0.735^***^	Y
H5c	SU → HSE	0.101	N

^a^ ***p < 0.001, **p < 0.01, *p < 0.05; ^b^ Y: support, N: not support

### Mediating effect test

Based on the three-step approach proposed by Baron and Kenney [58] and following the mediating effects testing procedure suggested by Zhonglin Wen et al. [59], the mediating effects of HSE between SS and SPB, PR between OS and SPB, and PR between SU and SPB is further tested with Model l4, a simple mediation expression in the SPSS macro prepared by Hayes [60], on the condition of controlling such demographic variables as gender, age, education, marital status and household registration category. Meanwhile, 95% confidence intervals are obtained with Bootstrap 5000 time sampling corrected. Tables 6 and 7 show the results of the sequential and Bootstrap tests, which suggests that OS, SS, and SU are all significant predictors of SPB. After the mediating variable PR is put in, the predictive effect of OS on SPB remains significant, but OS has a significant negative predictive effect on PR, so does PR on SPB. Besides, the Bootstrap 95% confidence intervals for both the direct effect of OS on SPB and the mediating effect of PR does not contain 0, which indicates that OS predicts SPB not only directly but also indirectly through the mediating effect of PR. Thus PR partially mediates the effect of OS on SPB. It’s the same case with SU. As for SS, after the mediating effect HSE is put in, the direct effect of SS on SPB is no longer significant though HSE has a significant positive predictive effect on SPB. It can also be found that the Bootstrap 95% confidence interval of the direct effect of SS on SPB contains 0, which indicates that SS can not predict SPB directly but through the mediating effect of HSE. Therefore, HSE fully mediates the effect of SS on SPB.

**Table 6:** Mediation effects test (sequential test results)

IV	M	DV	IV-DV	IV-M	(IV + M)-DV	
					IV	M
OS	PR	SPB	−0.215^***^	−0.429^***^	− 0.430^***^	− 0.502^***^
SS	HSE	SPB	0.116^**^	0.733^***^	−0.132	0.339^***^
SU	PR	SPB	−0.219^***^	−0.423^***^	− 0.431^***^	−0.501^***^

***p < 0.001, **p < 0.01

**Table 7:** Mediation effects test (BootStrap test results)

Pathway	Estimation	SE	95%CI
Indirect effect			
0S → PR → SPB	0.144	0.021	0.104 ~ 0.187
SS → HSE → SPB	0.163	0.038	0.093 ~ 0.238
SU → PR → SPB	0.210	0.030	0.153 ~ 0.270

CI: confidence interval

### Tests of mediating effects with moderation

**Fig. 2:**
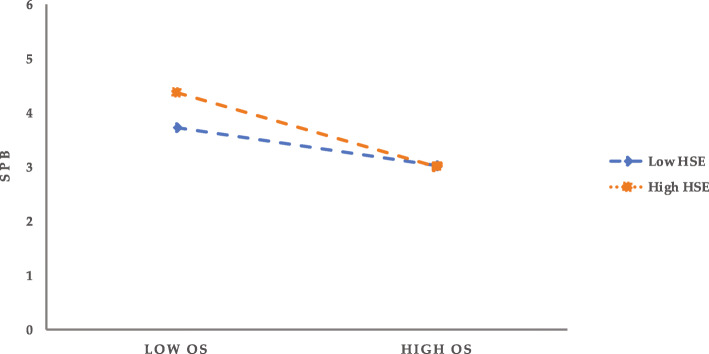
Regulatory role of HSE in the OS→PR → SPB pathway

**Fig. 3:**
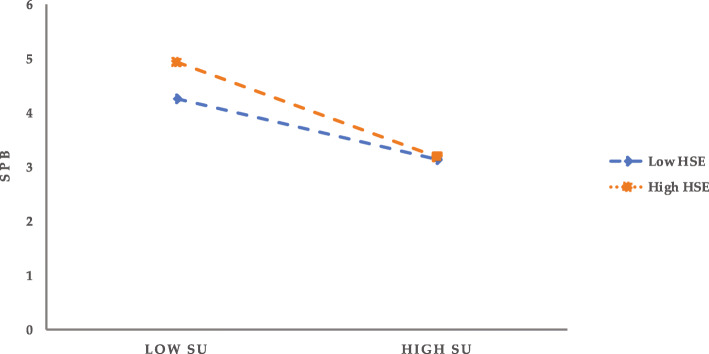
Regulatory role of HSE in the SU → PR → SPB pathway

The moderating effect of HSE on the mediating effect is further tested by means of Model14 in the SPSS macro developed by Hayes [60] (Model14 assumes that the second half of the mediating model is moderated) on the condition of controlling such demographic variables as gender, age, education, marital status, and household registration category. Meanwhile, 95% confidence intervals are obtained with Bootstrap 5000 time sampling corrected. The positive and negative one standard deviation of HSE is taken as the criteria for high and low HSE, and further simple slope analysis is performed (see Figs. 2 and 3). The results show that for the pathway OS→PR → SPB (see Fig. 2), PR is more likely to induce SPB in individuals with high HSE (M + 1SD) compared to those with low HSE (M-1SD). That is, when HSE is low, the predictive effect of PR on SPB is not significant (*bsimple* = − 0.08, t = − 1.76, *P* = 0.08); when HSE is high, the negative predictive effect of PR on SPB is significant (*bsimple* = − 0.52, t = − 14.42, P = < 0.001). For the path SU → PR → SPB (as seen in Fig. 3), PR has a significant negative predictive effect on SPB for individuals with high HSE (M + 1SD) (*bsimple* = − 0.51, t = − 14.65, *P* < 0.001); while for individuals with low HSE (M-1SD), PR also has a negative predictive effect on SPB, but its predictive effect is smaller (*bsimple* = − 0.10, t = − 2.22, *P* < 0.05), which indicates that the predictive effect of PR on SPB tends to increase gradually with the enhancement of HSE.

In addition, at the three levels of HSE, PR’s mediating effect on the relationship between SU and SPB also shows an increasing trend (see Table 8); that is, with the increase of individual HSE, SU is more likely to induce SPB by reducing public PR. It is worth emphasizing that, although the mechanism of the effect of PR on SPB in the pathway SU → PR → SPB differs for individuals with different levels of HSE, the negative effect of the former on the latter is statistically significant from the perspective of indirect effects. In other words, PR significantly and negatively affects SPB for both high HSE and low HSE individuals, and the mediating effect of PR in the OS-SPB relationship also tends to increase at all three levels of HSE (see Table 8).

**Table 8:** Bootstrap test results for mediating effects under HSE moderation

Pathway	Moderating variable (HSE)	Estimation	SE	95%CI
OS→PR → SPB	M-1SD	0.030	0.019	− 0.006 ~ 0.068
	M	0.116	0.019	0.078 ~ 0.154
	M + 1SD	0.201	0.028	0.146 ~ 0.255
SU → PR → SPB	M-1SD	0.055	0.026	0.007 ~ 0.109
	M	0.172	0.028	0.121 ~ 0.229
	M + 1SD	0.288	0.041	0.213 ~ 0.374

## Discussion

The purpose of this research is to explore the factors that influence SPB during the course of a pandemic, including PR, HSE, and SS. The moderating role of HSE and the controlling role of gender, age, marital status, education level, and household registration category are also identified. The results of the study facilitate our insight into the current status and influencing factors of SPB, and further help us identify critical control points for public self-health management behavior change, providing a theoretical basis as well as data support for the development of public self-health management programs to promote early intervention during the occurrence of a pandemic. A few findings are reported below.

First, among the three dimensions of social support, OS and SU have a significant impact on PR, while SS has no significant effect on PR. Thus, OS and SU are significant factors that influence people’s perceived risk of disease. In terms of importance, OS has a more significant impact with a path coefficient of 0.24, thus increasing OS is an effective means of reducing PR. The current study also supports the effect of SU, which is consistent with other studies’ results [27]. The effect of SS on PR is insignificant. The reason may be that the degree of disease risk perceived by individuals is mainly based on disease susceptibility and severity, which is a rational perception, whereas SS focuses on emotions.

Second, SS is in significant association with HSE. When individuals receive SS from various sources such as family, friends, government, they may become more confident in their ability to cope with risk. Thus, HSE can be effectively improved by increasing SS for individuals, which has been confirmed by previous studies [61]. Besides, the education degree has a positive influence on HSE. That is, the higher the education degree, the greater the ability of individuals to effectively manage their own health during the pandemic.

Third, as is hypothesized, SPB is significantly influenced by PR and HSE. At the same time, the mechanism by which individual perceived disease risk influences SPB is significantly and negatively moderated by HSE. When individuals perceive a higher risk of disease, they may be influenced by the psychological effects of anxiety and panic, which may contribute to the weakening of the individual’s intention to take precautionary behaviors. Then through the negative moderating effect of HSE, the individual’s intention for precautionary behavior will be enhanced. Specifically, as individuals’ perceived HSE increases, the mediating effect of PR in the association between SU and SPB and the mediating effect in the relationship between OS and SPB tend to increase. In other words, as individuals’ HSE increases, the OS and SU received by individuals are more likely to induce the adoption of SPB measures by reducing the level of PR. For the general population, individuals generally tend to understate their vulnerability to experiencing adverse life events like cancer, which is called optimism bias [62]. The low level of respondents’ PR in public in this study may be an example of it. The results of a study by Wise et al. suggest an optimism bias in the association between PR and SPB. Although most people are somewhat aware of the risks associated with COVID-19, they usually underestimate their personal risk compared with others [5]. In addition, gender is significantly associated with SPB, a finding that validates some of the previous findings [63, 64].

Fourth, mediating effects tests indicate that HSE significantly mediates the association between SS and intention to engage in SPB, and PR significantly mediates the relationship among OS, SU, and SPB. PR partially mediates the impact of OS on SPB, which, consequently, is indirectly influenced by SPB. The same results apply to SU. Thus, social support can indirectly influence SPB through PR and HSE.

## Implications and limitations

### Implications for theoretical research

Our findings provide some enlightenment for theoretical studies. First, against the background of the pandemic, this study empirically verifies that HSE and PR have significant effects on SPB and the moderating role of HSE in the association between PR and SPB. These findings have enriched the research on the relationship among PR, HSE, and SPB. Besides, the current study also examined the effects of social support factors on PR and HSE. Gender and educational attainment are significantly associated with variations in the RPA framework, which facilitates the clarification of future research on the application of the theory.

Secondly, the factors that influence SPB during a pandemic are examined in the current study. Although adequate findings have demonstrated that psychosocial factors are significant predictors of general protective health behaviors, the findings of the current research against the background of the pandemic highlight significant direct effects of PR and HSE on SPB, as well as indirect effects of OS, SU, and SS on SPB, which are relatively rare in previous studies concerned.

Third, HSE relates to education degree, and gender has a significant effect on SPB. These findings enrich the prediction of SPB during a pandemic.

### Enlightenment on practice

Our research findings have important significance for practical applications. There is growing evidence that public adoption of self-precautionary measures during a pandemic can have a powerful impact on controlling the spread of the virus and limiting its harmful consequences on physical health and the health care system [65, 66]. One of the most challenging tasks for the government is to encourage public participation in self-health management. Self-precautionary behavior is one of the main forms of self-health management activities. In the early stages of a pandemic, when effective vaccines are not available, the public may be advised to follow specific nonpharmacological measures proposed by the World Health Organization (WHO) and the Centers for Disease Control and Prevention (CDC).

On the one hand, predicting the public’s response to these measures and understanding the drivers of response can help governments better understand public perceptions and thus formulate specific public health plans. Our findings suggest that health self-efficacy is an essential factor influencing the public’s adoption of personal precaution measures during a pandemic. Therefore, the government can strengthen public health education and raise public awareness of epidemics and related preventive measures, thus increasing the public’s sense of self-efficacy in disease prevention and control. The public’s intention to adopt self-protection measures is enhanced by the perception that they are effective in reducing their own risk of infection and in overall epidemic control, confirmed by a study conducted in Spain [67]. On the other hand, as pointed out by Chaffee and Roser [40], individual behavior may be inhibited by the fear and anxiety induced by high-risk perception. Individuals’ perceived risk of illness has a negative effect on the adoption of precautionary measures. Therefore, the government can strengthen the construction of the social-psychological service system and focus partly on the formulation of policies related to general psychological guidance and policy implementation, like the formulation of the Technical Guidelines for Psychological Assistance Hotline. Second, Bish and Michie [63] concluded from their review that “high levels of trust in authorities and satisfaction with information received about the disease are associated with compliance with the prevention, avoidance, and management behaviors”, which has been confirmed by the actual outbreaks [68, 69]. Therefore, besides keeping the epidemic situation updated and increasing the transparency of the information on the epidemic, the government can also release the government’s efforts on the prevention and control of the epidemic. Through these objective supports, the public’s risk perception of the epidemic and anxiety are expected to be reduced to promote the public’s awareness of taking precautions.

### Limitations and future research

The current research has several limitations. First, the web-based online data collection by snowball sampling method in a short period explains the fact that the sample contains high ratio of young respondents as well as those with high education level. This may be a source of sample bias. The same research will be conducted in future studies by our testing a broader range of people, highly educated and non-highly educated individuals, for instance, to gain more valuable insights.

In addition, the research model developed in the current study is tested in China to examine the factors that influence the public’s precautionary behavior during a pandemic, so the findings of the study should be interpreted and applied with reservation due to the cultural differences between China and other countries.

## Conclusion

In the current study, an integrated theoretical research model based on the risk perception attitude framework and social support is developed, with the aim of examining the public’s intention of self-precaution during a pandemic. The current study may have two main contributions. First, it shows that PR and HSE have significant effects on SPB; HSE plays a moderating role in the relationship between SPB and PR. Thus, this study highlights the association between PR, HSE, and SPB against the background of the pandemic. Second, OS and SU significantly affect PR, whereas SS significantly affects HSE. Hopefully, these findings may enrich the researches related to the implications of social capital factors on PR and HSE.

## Data Availability

The datasets generated and/or analyzed during the current study are not publicly available due to limitations of ethical approval involving the anonymity but are available from the corresponding author on reasonable request.

## Abbreviations

COVID-19: coronavirus disease 2019
WHO: World Health Organization
MBIDs: mosquito-borne infectious disease
SPB: Self-precautionary behavior
HSE: Health self-efficacy
PR: Risk perception
OS: Objective support
SS: Subjective support
SU: Support utilization
CI: confidence interval
